# Unveiling the Photocatalytic Potential of BiAgOS Solid Solution for Hydrogen Evolution Reaction

**DOI:** 10.3390/nano14231869

**Published:** 2024-11-22

**Authors:** Oumaima Ben Abdelhadi, Majid El Kassaoui, Hajar Moatassim, Ahmed Kotbi, Mohamed Balli, Omar Mounkachi, Mustapha Jouiad

**Affiliations:** 1 Laboratory of Physics of Condensed Matter (LPMC), University of Picardie Jules Verne, Scientific Pole, 33 Rue Saint-Leu, CEDEX 1, 80039 Amiens, France; oumaima.benabdelhadi@um5r.ac.ma (O.B.A.); ahmed.kotbi@u-picardie.fr (A.K.); 2 Laboratory of Condensed Matter and Interdisciplinary Sciences, Physics Department, Faculty of Sciences, Mohammed V University in Rabat, Rabat 8007, Morocco; majid.elkassaoui@um5r.ac.ma (M.E.K.); hajar.moatassim@um5r.ac.ma (H.M.); 3 AMEEC Team, LERMA, International University of Rabat, Parc Technopolis, Rocade de Rabat-Sale, Rabat 11100, Morocco; mohamed.balli@uir.ac.ma; 4 Department of Mechanical Engineering, Faculté de Génie, Université de Sherbrooke, Sherbrooke, QC J1K 2R1, Canada; 5 College of Computing, Mohammed VI Polytechnic University, Ben Guerir 43150, Morocco

**Keywords:** oxychalcogenides, BiAgOS, hydrogen production, density functional theory (DFT), photocatalysis

## Abstract

The growing emphasis on green energy has spurred momentum in research and development within the field of photocatalytic materials, particularly for green hydrogen production. Among the most abundant oxides on Earth, oxychalcogenides stand out for their cost-effectiveness and ease of synthesis. In this context, we present an investigation of the potential use of BiAgOS as an efficient photocatalyst for hydrogen generation. Utilizing density functional theory and ab initio molecular dynamics (AIMD) simulations, we computed its physical properties and assessed its photocatalytic performance. Specifically, using Heyd–Scuseria–Ernzerhof corrections, our calculations yielded an appropriate electronic gap of ~1.47 eV necessary for driving the water-splitting reaction. Additionally, we obtained a very high optical absorption coefficient of ~5 × 10^5^/cm^–1^ and an estimation of hydrogen generation yield of ~289.56 µmol∙g^–1^. These findings suggest that BiAgOS holds promise for enabling the development of cheap, reliable, and highly efficient photocatalysts for hydrogen production.

## 1. Introduction

Technological advances and scientific progress have considerably improved the quality of human life [1]. However, this progress has also precipitated a surge in energy demand, particularly due to the expanding global population. Consequently, energy demands are reaching unprecedented levels [2]. Regrettably, much of the currently generated energy relies on fossil fuels, which have substantially contributed to greenhouse gas emissions, resulting in severe environmental challenges. In the pursuit of sustainable energy alternatives, green hydrogen emerges as a promising solution [3]. It is regarded as an inexhaustible clean energy carrier, boasting a high calorific value of 143 MJ/kg, three times superior to that of gasoline. Harnessing renewable resources to produce hydrogen via photoelectrochemical (PEC) water splitting by utilizing unlimited sunlight is considered one of the most desirable clean technologies. In addition to being water-stable and abundant on Earth, photoelectrode materials must exhibit a solar-to-hydrogen conversion efficiency of at least 10% to attain commercial viability [4]. Various materials have been evaluated as photocatalysts, yielding varying degrees of success, including TiO_2_, SrTiO_3_, NaTaO_3_, WO_3_, Fe_2_O_3_, BiVO_4,_ and Ta_3_N_5_ [5,6,7,8,9,10,11]. These materials have not transitioned to the manufacturing stage due to their high costs. Therefore, given their significant social and industrial implications, ongoing efforts are required to propose alternative photocatalysts. In this regard, it is worthwhile to explore oxychalcogenides due to their exceptional physical properties and cost-effectiveness. This class of oxides is straightforward to synthesize and exhibits thermal stability. Moreover, unlike other oxides, oxychalcogenides offer greater flexibility in tailoring their bandgap and electrical properties. Recent progress in exciton manipulation in metal chalcogenides underscores the potential of heterojunction approaches for enhancing hydrogen production efficiency, which aligns with our exploration of the BiAgOS solid solution [12].

Initially, the focus was on developing oxychalcogenides of the form LnMOX (Ln = Bi, Ce, Dy, La; M = Cu, Ag; X = S, Se, Te) [13], then turned to Bi-based isostructural analogs such as BiCuOX (X = S, Se), which was later shifted to Bi-based isostructural compounds, namely BiCuOX (X = S, Se), first developed in the 1990s. In particular, BiAgOX phases have garnered extensive attention for their thermoelectric properties, favorable optical response, and catalytic activities due to their low intrinsic thermal conductivity and high electronic conductivity [14]. This phenomenon is attributed to the coexistence of ionic oxide anions and more covalent chalcogenide anions, resulting in a distinct structural chemistry. It is widely acknowledged that quaternary oxychalcogenides typically adopt structures where oxide and chalcogenide anions are segregated. Each type of anion preferentially bonds to a specific cation, with non-polarizable cations tending to coordinate with smaller oxide anions and more polarizable cations preferring larger chalcogenide anions [15]. The low dimensionality of the structure can yield highly anisotropic electronic band structures and intriguing electronic properties. Furthermore, the high ionic interactions within the oxide blocks promote low thermal conductivity, while the covalent nature of the chalcogenide layers supports high carrier mobility.

Herein, in this context, we investigate the BiAgOS compound as a promising photocatalyst for water-splitting reactions and hydrogen production, employing density functional theory calculations. Our analysis encompasses electronic properties, such as electronic band structure and density of states, alongside optical properties, such as variations in refractive index, extinction coefficient, reflection, and absorption. Additionally, we evaluate the thermoelectric properties of BiAgOS as an alternative avenue for power generation. The assessment of BiAgOS considers several factors, including band gap, alignment of band edges with water redox potentials relative to pH, and hydrogen yield.

## 2. Computational Methods

First-principles calculations based on density functional theory (DFT) are performed using the Quantum espresso code. Following the optimization of the structure, a self-consistent calculation is performed to extract physical properties using NORMCONS pseudopotentials. To enhance band gap calculation accuracy, the Heyd–Scuseria–Ernzerhof (HSE) exchange-correlation functional was employed, while the Gygi–Baldereschi method was used to deal with the divergence of the exact exchange energy at small G-vectors. Prior to the calculation, both cell parameters and atomic positions are relaxed to obtain an equilibrium structure. Structural convergence is attained using a cutoff energy of 70 Ry, with a 3 × 3 × 3 Monkhorst–Pack *k* mesh for structural relaxation and 12 × 12 × 12 for the calculation of other properties. The Methfessel–Paxton smearing has been taken into account in our calculations. The value of the smearing width used is 0.02. Ion and electron relaxation convergence is achieved at approximately ~10^–6^ eV. The optical properties of BiAgOS are calculated using the YAMBO code [16]. Ab initio molecular dynamics (AIMD) calculations begin with the preparation of the system, where the initial structure is defined by a 3 × 3 × 1 supercell of BiAgOS. The simulation conditions are set in the NVT ensemble (constant number of particles, volume, and temperature) at a temperature of 300 K. At each time step, the total energy of the system and the forces acting on the atoms are calculated using DFT. These forces are then used to update the positions and velocities of the atoms at each time step (in the order of a femtosecond, 1 fs) using an integration algorithm. This process is iterated over a total duration of 7 ps to obtain the atomic trajectories. The analysis of these trajectories allows the assessment of thermal stability: the absence of significant structural deformations during the simulation indicates thermal stability at the simulated temperature [17]. Thermoelectric properties were calculated using the Boltztrap package [18] based on band structures obtained by ab initio calculations.

Complex interband dielectric functions, *ε_inter_*(ω), are conducted in the random-phase-independent particle approximation, i.e., taking into account only direct transitions from occupied to unoccupied Kohn–Sham orbitals. As optical data on hydrides often originate from micro- and nanocrystalline samples with variable crystallite orientation, the direction-averaged dielectric function is considered the most relevant quantity. The intraband dielectric function, *ε_intra_*(*ω*), is derived from the free electron plasma frequency *ω_p_*, as follows:

(1) $$\varepsilon \left(\omega \right)=\varepsilon_{intra}^{\left(1\right)}+i\varepsilon_{intra}^{\left(2\right)}$$

(2) $$\varepsilon_{intra}^{\left(1\right)}=1-\frac{\omega_{P}^{2}}{\omega^{2}+\gamma^{2}}$$

(3) $$\varepsilon_{intra}^{\left(2\right)}=\frac{{\gamma \omega }_{p}^{2}}{\omega^{3}+{\omega \gamma }^{2}}$$

The following formula is used to obtain the plasma frequency *ω_p_* as an integral over the Fermi surface:

(4) $$\omega_{p\left(\alpha \beta \right)}^{2}=\frac{4\pi e^{2}}{V\hbar^{2}}\sum_{n,k}2g_{k}\frac{\partial f(\epsilon_{nk})}{\partial \epsilon }(e_{\alpha }\frac{\partial (\epsilon_{nk})}{\partial \epsilon k})(e_{\beta }\frac{\partial \left(\epsilon_{nk}\right)}{\partial \epsilon k})$$

where *f(*ε*_nk_)* is the occupation function, and *g_k_* is the weight factor. We employ directionally averaged values once more. Additional information on the plasma frequency calculation is available in Ref. [19]

The optical constants, including the refractive index (*n*), extinction coefficient (*κ*), absorption (*A*), and reflection (*R*), are computed using the following standard expressions:

(5) $$A=1-e^{\frac{-k\omega d}{c}}$$

(6) $$R=\frac{{\left(n-1\right)}^{2}+k^{2}}{{\left(n+1\right)}^{2}+k^{2}}$$

where *ε*^(1)^ and *ε*^(2)^ are the real and the imaginary components of *ε*, *d* is the slab thickness, and *c* is the speed of light in vacuum [20].

The BoltzTrap package is based on the linearized Boltzmann transport theory. Here are the key equations used [18]:

(7) $$S_{\alpha \beta }\left(T,\mu \right)=\frac{1}{eT\Omega \sigma_{\alpha \beta }(T,\mu )}\int \sigma_{\alpha \beta }\left(\varepsilon \right)\left(\varepsilon -\mu \right)\left[-\frac{\delta f_{0}(T,\varepsilon ,\mu )}{\delta \varepsilon }\right]d\varepsilon$$

(8) $$\sigma_{\alpha \beta }\left(T,\mu \right)=\frac{1}{\Omega }\int \sigma_{\alpha \beta }(\varepsilon )\left[-\frac{\delta f_{0}(T,\varepsilon ,\mu )}{\delta \varepsilon }\right]d\varepsilon$$

(9) $$k_{\alpha \beta }\left(T,\mu \right)=\frac{1}{e^{2}T\Omega }\int \sigma_{\alpha \beta }\left(\varepsilon \right){\left(\varepsilon -\mu \right)}^{2}\left[-\frac{\delta f_{0}(T,\varepsilon ,\mu )}{\delta \varepsilon }\right]d\varepsilon$$

The transport distribution tensor elements, in this case, are determined by Fourier interpolation of the band structure, denoted by *σ_αβ_*. The tensor indices are denoted by *α* and *β*, while the cell volume, chemical potential, Fermi distribution function, and absolute temperature are represented by *Ω*, *μ*, *f*_0_, and *T*, respectively.

The crystal structure and the band structure on a uniform grid are the input data required to execute the BoltzTraP function. The Boltzmann equation can be solved with the BoltzTraP algorithm [21].

(10) $$\frac{\partial }{\partial t}f+\vec{\upsilon }\frac{\partial }{\partial \vec{r}}f+\frac{eE}{\hbar }\frac{\partial }{\partial \vec{k}}f={\left(\frac{\partial f}{\partial t}\right)}_{scattering}$$

where *k* is the wave vector, *v* is the particle’s velocity vector, and *f* is the distribution function.

The BoltzTraP code interpolates a band structure and completes the necessary integrations to solve the Boltzmann equation [22].

The standard water reduction and oxidation at the point of zero charge solution was calculated as below:

(11) $$E_{CB}^{pH=0}=\chi +E_{0}-0.5\cdot E_{g}$$

(12) $$E_{VB}^{pH=0}=E_{CB}^{pH=0}+E_{g}$$

(13) $$E_{\left(CB,VB\right)}^{pH}=E_{\left(CB,VB\right)}^{pH=0}-0.059\cdot pH$$

where *χ* is the Mulliken electronegativity, *E_0_* (−4.5 eV) is the reference potential used to link the redox level to the vacuum, and *E_g_* is the electronic band gap energy for BiAgOS calculated using HSE approximation [23]. Equation (13) allows us to obtain band edge potentials as a function of pH. This equation incorporates the Nernst relation to account for the pH dependence [24].

Many materials are only visible on phase diagrams at elevated temperatures, making it crucial to account for thermal effects to predict their stability under both ambient and high-temperature conditions. The role of entropy in thermodynamics is evidenced by Gibbs free energy equation, as follows:

(14) G=U+PV−TS=H−TS 

where *T* is the temperature and *F* = *U* − *TS* denotes the Helmholtz free energy. The balance between the enthalpy and the entropy is key in many crystalline processes, including defect formation and the miscibility limits of the solid solutions. Enthalpy-entropy compensation is a general characteristic of chemical reactions, particularly in phase transitions [25].

## 3. Results and Discussion

### 3.1. Structure, Stability, and Electronic Properties

**Figure 1:**
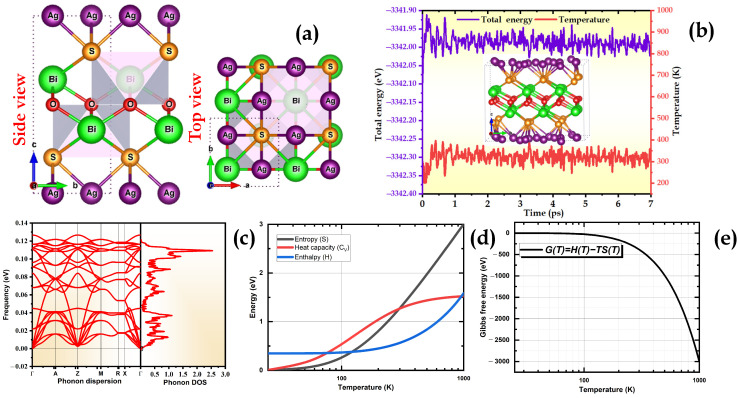
(**a**) Top/side view of BiAgOS crystal after relaxation, (**b**) top snapshot with the corresponding variation of the total energy between 0 and 7 ps during the AIMD simulations at 300 K, (**c**) the phonon spectra and PhDOS, (**d**) variation of entropy S, heat capacity C_V_, and enthalpy H as a function of temperature, and (**e**) variation of Gibbs free energy as a function of temperature.

The structure of BiAgOS belongs to the ZrSiCuAs-type structures with a P4/nmm tetragonal space group [26]. There are two different layers in a unit cell: a positively charged oxide layer (Bi_2_O_2_^2+^) and a negatively charged chalcogenide layer (Ag_2_S_2_^2−^). Along the crystallographic c-axis, the layers are stacked in the sequence 2O-Bi-S-2Ag. The optimized geometry of BiAgOS is displayed in Figure 1a.

The obtained optimal equilibrium parameters are a = b = 3.99 Å and c = 9.26 Å. The Ag-S and Bi-O bond lengths are 2.68 Å and 2.34 Å, respectively, which is consistent with reported values (Table 1).

The calculations were performed on a primitive BiAgOS cell containing eight atoms (each element contains two atoms). In the following step, we will examine the structural and thermal stability of BiAgOS in detail by performing AIMD calculations at room temperature (300 K) with a total time scale of 7 ps. Overall, Figure 1b clearly shows that the total energy fluctuation of our system is small, suggesting that the BiAgOS material is thermally stable under realistic experimental conditions.

To further confirm the dynamic stability of our system, we utilized the Phonopy code to assess its vibrational properties. The results indicate that the vibrations occur at positive frequencies, suggesting high stability. Importantly, the absence of imaginary frequencies in the phonon spectrum confirms the dynamic stability of our system. Additionally, the Gibbs free energy is a key indicator for understanding and optimizing the catalytic performance of our system for both oxygen evolution reaction (OER) and hydrogen evolution reaction (HER) during water splitting driven by solar energy. The Gibbs free energy change during the reaction allows for determining the thermodynamic feasibility and efficiency of the reaction on the catalyst’s active sites. By lowering the Gibbs free energy in OER and HER, the energy barrier required to initiate and sustain these reactions is reduced, thereby enhancing the catalytic efficiency. The reduction in Gibbs free energy makes the reactions thermodynamically more favorable, which is crucial for developing efficient, durable, and cost-effective catalysts for hydrogen production via water splitting [29]. Figure 1d illustrates the evolution of entropy (S), heat capacity (C_V_), and enthalpy (H) of the material as a function of temperature. The increase in enthalpy with temperature indicates that more energy is stored as internal heat at higher temperatures. The heat capacity C_V_ also increases with increasing temperature due to changes in the material’s internal energy modes, making the system more thermally active. In Figure 1e, a significant decrease in Gibbs free energy at 100 K indicates that the process becomes thermodynamically more favorable as the temperature rises, which is a crucial consideration for catalytic applications, such as hydrogen evolution (HER).

**Figure 2:**
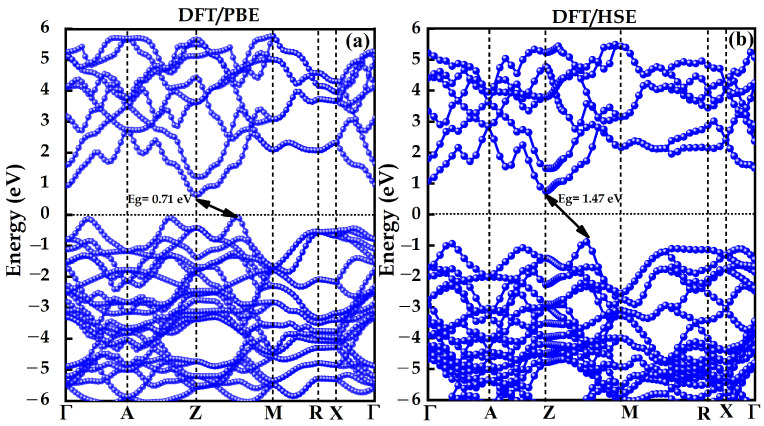
DFT computed band structures of BiAgOS using (**a**) PBE approximation and (**b**) HSE approximation.

Furthermore, the band gap energy is one of the important characteristics of a photocatalyst material, as it needs to remain sufficiently separated to facilitate the water-splitting reaction under light excitation. The minimum theoretical bandgap energy range required for photocatalytic materials to drive the reaction is approximately 1.23 eV. According to our simulation, the electronic structure reveals an indirect transition upon excitation at the band gap. The density of states (DOS) and band positions were initially determined using the PBE approximation, revealing a bandgap of 0.71 eV, shown in Figure 2a. This underestimation of the bandgap was corrected using the HSE approximation, as depicted in Figure 2b. The band gap calculated for BiAgOS using the HSE06 function and spin–orbit coupling is approximately 1.47 eV.

**Figure 3:**
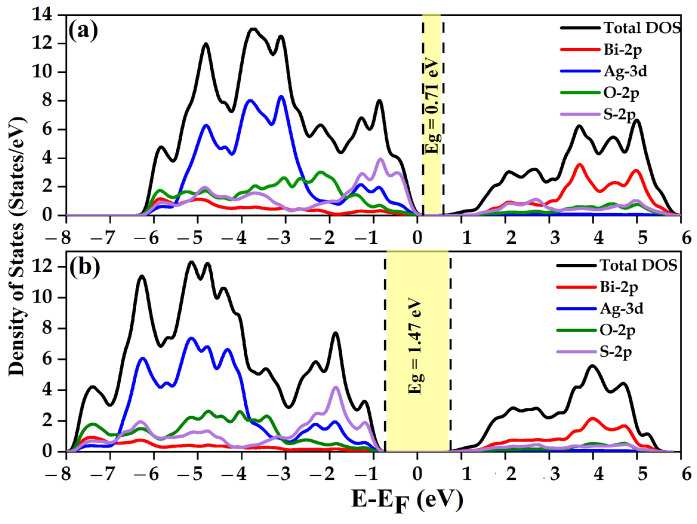
DFT computed the total and partial DOS of BiAgOS using (**a**) PBE approximation and (**b**) HSE approximation. The Fermi energy level (*E_f_*) is set to 0 eV.

The density of states (DOS) curve gives insight into the electronic structure of materials, as shown in Figure 3a,b.

In the case of Ag atoms interacting with S atoms, a strong hybridization occurs. The d-orbitals of Ag and the p-orbitals of S overlap, resulting in hybridized states in both the valence and conduction bands. These hybrid states exhibit a blend of Ag and S characteristics, impacting binding and material stability. Similarly, Bi atoms interact with oxygen atoms, leading to a robust hybridization between both atoms. The DOS curve reflects these hybridizations, displaying peaks near the valence band and a decrease near the conduction band. Overall, the DOS curve elucidates the interaction between Ag and S orbitals, as well as Bi and O, which influences the material’s properties and behavior. Peaks indicate regions of high electron density, indicating the availability of states for electronic occupation, while valleys represent energy gaps where electronic states are less likely. The hybridization between Ag-d and S-p orbitals, as well as Bi-d and O-p orbitals, effectively alters the electronic structure of the material, impacting its electrical conductivity, optical properties, and other essential characteristics.

### 3.2. Optical Properties

**Figure 4:**
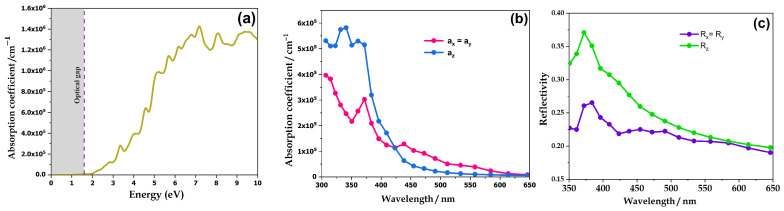
Computed optical properties of BiAgOS: (**a**) absorption as a function of energy, (**b**) absorption coefficient, and (**c**) reflectivity.

The response of electrons to a wave in a material is characterized by the complex dielectric constant ε(ω), where the imaginary component reflects the absorption of the material, while the real component is associated with the polarization of the medium. Using Equations (5) and (6), we calculated the absorption A, as well as the reflectivity spectrum R. The results are shown in Figure 4.

Figure 4a depicts the variation of the absorption coefficient α, which increases with increasing energy, reaching a maximum value of ~7 eV. Moreover, the optical gap is found to be ~1.78 eV according to the HSE approximation. BiAgOS absorbs more up to 5.8 × 10^5^ cm^–1^ in the ultraviolet regions and 1.8 × 10^5^ cm^–1^ of visible light, which is consistent with the experimental value of 1.4 × 10^4^ cm^–1^ reported [26].

The reflectivity spectrum R, shown in Figure 4c, describes the amount of radiation reflected at the surface of the material, with a thickness allowing the reflectance to remain constant despite increasing thickness. The reflectivity spectrum is influenced by the bandgap energy. A high energy bandgap results in a low reflection coefficient, consequently facilitating light absorption or transmission through the material. For BiAgOS, we can clearly see that it exhibits noticeable reflectivity, particularly within the ultraviolet to infrared wavelength range, as reported elsewhere [31,32].

### 3.3. Thermoelectric Properties

To obtain an optimized thermoelectric material, it is imperative to take into account various competing properties such as high Seebeck coefficient, high electrical conductivity, and low thermal conductivity. However, these parameters are not independent. Previous works have explored phonon engineering to improve phonon scattering and increase the figure of merit without impacting the electronic part. The thermoelectric material Bi_2_Te_3_ was proposed as a thermocatalyst capable of generating hydrogen peroxide (H_2_O_2_) under small differences in surrounding temperature [33]. When a temperature difference is applied, negative charges migrate from the hot to the cold side of the material, creating a potential between its hot and cold ends. This thermocatalytic property is specific to thermoelectric materials capable of separating electrons and holes under the effect of a thermal gradient. The potential induced by this thermal gradient in thermoelectric materials paves the way for efficient thermocatalysts, meaning photocatalysis at high temperatures. Our study focuses on the electronic component of thermal conductivity. In our study, we are interested in calculating thermoelectric properties to determine whether our material is a good thermocatalyst. This is to facilitate the stimulation of chemical reactions, not for the production of electricity, but to generate a potential from a temperature difference, thus promoting a photoelectrochemical reaction or a redox reaction for the production of H2. However, not all good thermoelectric materials are necessarily good thermocatalysts. Besides the fundamental factors influencing the thermocatalytic performance of thermocatalysts, including band alignment, charge carrier mobility, and stability, a high Seebeck coefficient is crucial for enhanced thermocatalytic activity. Specifically, a higher Seebeck coefficient results in a higher thermoelectric voltage, facilitating charge separation and promoting thermocatalytic reactions.

**Figure 5:**
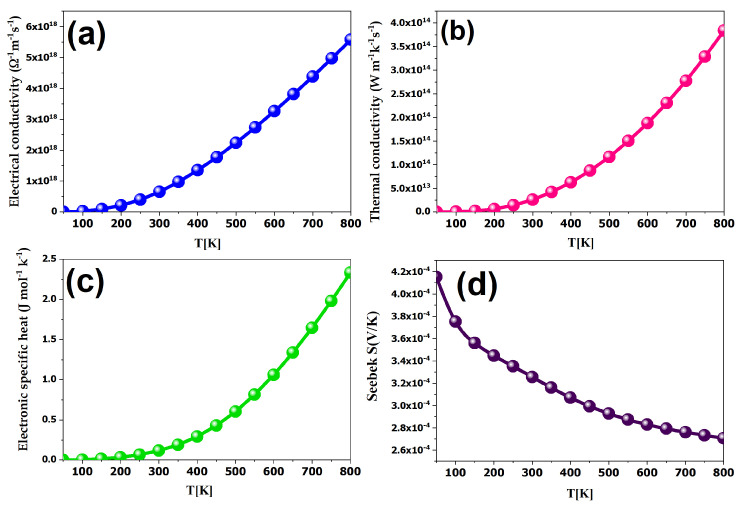
Computed thermoelectric properties of BiAGOS: (**a**) electrical conductivity, (**b**) thermal conductivity, (**c**) electronic specific heat, and (**d**) Seebeck coefficient as a function of temperature.

Parameters such as the Seebeck coefficient, electrical conductivity, and the electronic part of thermal conductivity are directly influenced by the number of charge carriers, creating significant complexity. Increasing carrier concentration has concomitant effects on the electrical and electronic parts of thermal conductivity while simultaneously decreasing the Seebeck coefficient. Our main objective is to determine the optimal values of these strongly correlated parameters with constant carrier concentration. Our study on BiAgOS examines the Seebeck coefficient (S), electrical conductivity over relaxation time (σ/τ), and electrical conductivity over relaxation time (κe/τ) over a temperature range from 100 K to 800 K, as shown in Figure 5.

The results demonstrate promising Seebeck coefficients, reaching 330 (µV/K) at room temperature, which is higher than 202 (µV/K), which is the optimal Seebeck coefficient value for a good thermoelectric material [34] and also higher than 244 (µV/K) [35]. As can be seen in Figure 5, there is an increase in the electrical conductivity of BiAgOS with increasing temperature, which is favorable to thermoelectric performance, yielding a higher power factor. Despite the increase in electron thermal conductivity with temperature, it remains lower than electron electrical conductivity, leading to high figure-of-merit values for both materials. Finally, it is worth pointing out that although this paper does not investigate the lattice’s thermal conductivity, the values of the electronic parameters suggest that BiAgOS could also be a viable candidate for thermoelectric applications. It is worth noting that the excellent thermoelectric properties of BiAgOS would enhance its thermal stability during water-splitting reactions, particularly under prolonged heating conditions.

### 3.4. Photocatalytic Properties

Thermodynamically, an effective photocatalyst must have $E_{CB}^{pH=0}\;$ positioned above the water reduction (E_H+/H_2__ = 0 eV), which implies that e-CB can engage in a reaction with H^+^ to generate H_2_ gas, as follows:

2H^+^ + 2e^−^ →H_2_ (15)

And $E_{VB}^{pH=0}\;$ positioned below the water oxidation level (E_O_2_/H_2_O_ = 1.23 eV). Consequently, *h*^+^ VB has the capability to drive water reduction reaction, resulting in the production of oxygen gas, as follows [36]:

H_2_O + 2*h*^+^→2H^+^ + 1/2 O_2_ (16)

**Figure 6:**
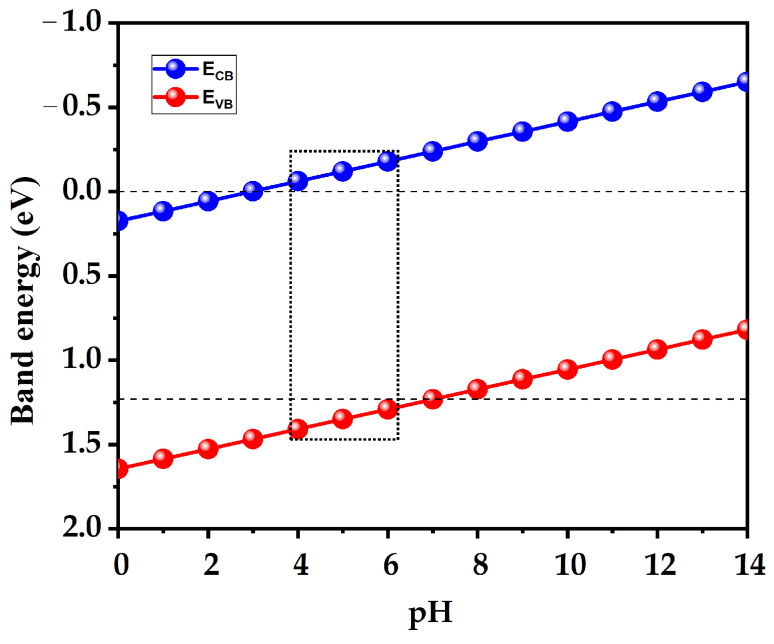
Variation in conduction band energy (blue) and valence band energy (red) as a function of pH.

So, it is important to know the E_CB_ to determine whether the conduction band electrons have sufficient reducing power for the desired photocatalytic reactions. The E_CB_ must be more negative than the redox potential of the target reaction for efficient electron transfer [37]. In our study, BiAgOS, under vacuum conditions, does not produce hydrogen from photocatalytic water splitting due to the position of its band edge. However, adjusting the pH of the solution can shift the band edge alignment, balancing the oxidizing and reducing potentials and making it compatible with the redox potential of H^+^/H_2_ and O_2_/H_2_O. As shown in Figure 6, both the oxidation and reduction potentials have shifted from their initial values, making BiAgOS suitable for hydrogen production in photocatalytic applications, particularly in acidic solutions with a pH range between 4 and 6 (Figure 6). To assess the amount of hydrogen yielded by our compound, we computed the effective electron mass ratio (m_e_*/m_0_), the effective density of states N_c_^+^ (cm^−3^), and the charge carrier density (n) of BiAgOS [38]. It is important to note that the me*/m_0_ value was derived from the computed band structure by fitting the CB minimum with a paraboloid. For the BiAgOS system, we obtain a m_e_*/m_0_ value equal to ~4.77.

$N_{c}=2.5\times 10^{19}\times {\left(\frac{m^{*}}{m_{0}}\times \frac{T(K)}{300}\right)}^{3/2}=2.58\times 10^{20}{cm}^{-3}$, where n = Nc ~ 2.58 × 10^20^ cm^−3^

1 mole = $6.023\times 10^{23}$ H_2_; hence, $x_{mole}=2.14\times 10^{-4}mole/{cm}^{3}$ (given H_2_O + 2e = H_2_)

$$H_{2yield}=\frac{2.14\times 10^{-4}}{\frac{m\left(g\right)}{N_{a}\times Volume({cm}^{3})}}=289.56\;µmol/g$$

Our reported yield of ~289.56 µmol∙g^−1^ is higher than the reported one ~150 µmol∙g^−1^, obtained for ZnO:Al/MAPbI_3_/Fe_2_O_3_ composite [39]. To calculate the solar-to-hydrogen (STH) efficiency, we use the following equation:

$$STH=\frac{R_{H_{2}}\times ∆G}{P_{sun}\times S}\times 100\%$$

where is $R_{H_{2}}$ our calculated hydrogen yield of 289.56 µmol/g, ∆G is the calculated Gibbs free energy, $P_{sun}$ is the sunlight power and S the exposed area of the catalyst. We assume typical 1-sun (1 AMG) conditions with light intensity at 100 mW/cm^2^ and an exposure time of 1 h. Based on this, we estimate a theoretical STH efficiency limit of approximately 19.06%. However, considering the oxygen vacancies and the overpotential, the actual STH efficiency is expected to be much lower than this theoretical prediction”.

The experimental feasibility of synthesizing BiAgOS using various techniques has been demonstrated. This material has been successfully synthesized using hydrothermal synthesis [29,40], solid-state reaction [14], as well as the ball-milling method [41]. Although these studies were conducted for other applications, such as photoelectronic devices [42], photoconversion [29], and the use of BiAgOS–PEG nanoparticles as a semiconductor photosensitizer for cancer treatment, due to their excellent photothermal and photosensitive properties, as well as their broad optical absorbance [43], the results confirm that BiAgOS synthesis is both feasible and adaptable. This opens promising perspectives for numerous applications in the field of advanced materials, including hydrogen production [42,43,44,45].

Our findings suggest that the intrinsic material BiAgOS exhibits promising characteristics as a photocatalyst. Firstly, its efficient light absorption in the ultraviolet and visible range implies its capability to utilize a broad spectrum of wavelengths for initiating photocatalytic reactions. Moreover, the data indicate a notable yield in hydrogen production. These observations propose that BiAgOS holds the potential to play a significant role in solar-to-chemical energy conversion processes. In summary, the outcomes of this investigation suggest that BiAgOS has the potential to serve as an effective catalyst in photocatalysis, thereby laying the groundwork for future applications in solar energy conversion.

## 4. Conclusions

We conducted a comprehensive examination of BiAgOS properties utilizing density functional theory calculations, capitalizing on the remarkable advancements achieved by the DFT community in simulating semiconductor properties. These advancements stem from novel functional developments like HSE06, along with new property implementations such as optoelectronic property calculations using this approximation. These calculations enabled us to explore the structural, electronic, optical, thermoelectric, and photocatalytic characteristics of this promising material. Analysis of the results revealed the electronic structure of BiAgOS, unveiling an indirect photon absorption transition with a band gap of 1.47 eV. Additionally, we observed a remarkably high optical absorption coefficient, approximately 10^5^ cm^−1^, in the visible and ultraviolet regions of the solar spectrum. Our findings also showcased promising Seebeck coefficients, reaching 330 µV∙K^−1^ at room temperature, along with an increase in electrical conductivity. Although the electrical conductivity was lower than thermal conductivity, it enhanced thermoelectric performance and power factor. Moreover, under visible light irradiation, we observed a hydrogen production rate of up to 289.56 µmol∙g^−1^, a notable achievement for an intrinsic material.

## Figures and Tables

**Table 1:** The calculated lattice parameters of BiAgOS using HSE approximation compared to experimental ones.

	*A = b* (Å)	*c* (Å)	Ref.
DFT calculations	3.92	9.218	This work
	3.99	9.26	[27]
	3.91	9.23	[28]
	3.936	9.148	[29]
Experiment	3.913	9.228	[30]

## Data Availability

The original contributions presented in the study are included in the article. Further inquiries can be directed to the corresponding author.
